# Predictors of workplace violence among ambulance personnel: a longitudinal study

**DOI:** 10.1002/nop2.38

**Published:** 2015-11-04

**Authors:** Peter G. van der Velden, Mark W.G. Bosmans, Erik van der Meulen

**Affiliations:** ^1^ INTERVICTTilburg UniversityWarandalaan 25037 ABTilburgThe Netherlands

**Keywords:** Aggression, ambulance personnel, coping, handling rules, nurses, organizational stress, personality, trauma, workplace violence

## Abstract

**Aim:**

To examine predictors of repeated confrontations with workplace violence among ambulance personnel, the proportion of exposure to potentially traumatic events that are aggression‐related and to what extent personnel was able to prevent escalations. Although previous research assessed the prevalences among this group, little is known about predictors, to what extent PTE's are WPV‐related and their abilities to prevent escalations.

**Design:**

A longitudinal study with a 6 months' time interval (*N *=* *103).

**Methods:**

At T1 demographics, workplace violence and potentially traumatic events in the past year, mental health, personality, handling of rules, coping and social organizational stressors were assessed. Confrontations with aggression were also examined at T2.

**Results:**

Multivariate logistic regression analyses showed that only problems with superiors independently predicted repeated verbal aggression and that only the (absence of the) ability to compromise very easily predicted repeatedly being on guard and repeatedly confronted with any form of aggression. Due to very low prevalences, we could not examine predictors of repeated confrontations with physical aggression (*N *=* *5) and serious threat (*N *=* *7). A large majority reported that in most workplace violence cases they could prevent further escalations. About 2% reported a potentially traumatic event in the year before T1 that was WPV related and perceived as very stressful.

## Introduction

Health services personnel are at risk of being confronted with aggression from patients, clients or their relatives (Camerino *et al*. 2008, Iennaco *et al*. 2013, Magnavita 2014, Nolan *et al*. 1999). This so‐called workplace violence (WPV) varies from physical aggression, serious threat and verbal aggression aimed at the personnel and situations where personnel are on guard, because they were afraid that people might become aggressive, although no single definition of WPV exists (cf. Beech & Leather 2006). WPV may have adverse effects on the mental health of affected personnel (van der Velden & Herpers 1994, Winstanley & Whittington 2002, van der Ploeg & Kleber 2003, Inoue *et al*. 2006, Magnavita 2014). Previous studies have shown that ambulance personnel are not exempted from WPV. For example, in the studies of Bigham *et al*. (2014), Boyle *et al*. (2007) and Petzäll *et al*. (2011) the 12‐month prevalences of WPV ranged from 66·0‐87·5%. These prevalences indicate that WPV needs serious attention since (besides mental health effects) coping with aggression and violence is not considered the primarily task of ambulance personnel, in contrast to for example police officers (Rabe‐Hemp & Schuck 2007, van der Velden *et al*. 2010),

### Background

Measures or the development of measures to minimize the risk of WPV will be dependent on factors related to WPV, such as those related to the characteristics of: (a) the patients, clients or their relatives; (b) organizational or situational issues and (c) the characteristics of the victimized ambulance personnel. For example, Grange and Corbett (2002) showed that substance abuse and psychiatric disorders among patients, were associated with a heightened risk for WPV among paramedics. Other studies demonstrated that work‐related factors of working in night shifts, work dissatisfaction and nursing discipline were related to WPV (Arnetz *et al*. 1996, Zeng *et al*. 2013). Bilgin (2009) showed that conflict management styles of more help seeking, being more sociable, being more tolerant and being more trusting were negatively associated with WPV among a sample of Turkish mental health nursing students. With respect to the demographical characteristics of personnel, previous cross‐sectional studies found mixed results with respect to gender. While Arnetz *et al*. (1996) and Zeng *et al*. (2013) showed an increased risk for males, Bigham *et al*. (2014) showed an increased risk for females. The study by Bigham *et al*. (2014), however, was conducted among paramedics specifically. The cross‐sectional study by Boyle *et al*. (2007) also found a heightened risk for females, but only for sexual harassment and abuse among paramedics. In addition, in the cross‐sectional studies of Bigham *et al*. (2014) and Zeng *et al*. (2013) younger and less experienced nurses were more at risk of WPV.

For non‐stable characteristics, in contrast to stable characteristics such as gender and age, longitudinal studies on predictors on WPV are warranted to prevent the ‘egg‐chicken’ problem (cf. Magnavita 2014). The study by Magnavita (2014) examined WPV among healthcare workers over a period of 6 years and found job strain to be predictive of WPV, whereas WPV itself explained job strain thereafter. Earlier, Hogh and Viitasara (2005) conducted a systematic review on risk factors on WPV including WPV in the health services. Although no ambulance personnel studies were included in this review, findings showed that being male, less experienced and having interpersonal conflicts were predictive of WPV. According to this review, age was inconsistently related to WPV across studies with some studies reporting being younger as a risk factor, whereas other reported being middle‐aged as the risk factor. The included populations are diverse as they range from general health care to specific psychiatric care and thereby these populations might not be comparable in terms of associations with WPV. The most important prospective risk factor identified in this review was earlier WPV: WPV predicts future WPV (cf. Hogh *et al*. 2008). An explanation for this finding is the possible vicious circle between being victimized by aggression resulting in anger and hostility increasing the risk for escalating behaviour in future events instead of de‐escalating behaviour (cf. Hogh *et al*. 2005), which is in line with the study of Magnavita (2014).

To what extent (mental) health problems, that may diminish/influence work performance, is predictive of repeated confrontations with WPV among ambulance personnel is unknown. In addition, research had shown that coping self‐efficacy related to potential traumatic events such as WPV, is predictive of the development of postevent mental health problems (Luszczynska *et al*. 2009, Bosmans & van der Velden 2015). Higher levels of coping self‐efficacy are associated with lower levels of mental health problems or stress symptoms at a later stage. To the best of our knowledge, to date no study examined the role of coping self‐efficacy in repeated confrontations with WPV: does the perceived ability to cope with potentially traumatic events such as WPV influence the prevalence of repeated confrontations? The same question arises with respect to the coping style ‘seeking social support’: does seeking social support in critical situations such as WPV reduce the risk of WPV? In line with the last questions (ability to cope with events and coping), little is known about to what extent ambulance personnel is able to prevent further escalations during these incidents and how often do they use aggression themselves.

In sum, with respect to ambulance personnel, little is known about prospective predictors of WPV and especially of repeated confrontations with WPV. Aim of this study was to help to fill this gap of information.

When presenting prevalences of WPV among ambulance personnel, or mental health problems following these events, they are often primary viewed as ‘victims’. However, this may distract us from their abilities to de‐escalate aggressive behaviour used against ambulance personnel. We are not aware of any study examining this aspect among ambulance personnel.

## The study

### Aims

The aim of this study was to fill this gap of information. Central research questions are: (1) What are the prevalences of repeated confrontations with aggression towards ambulance personnel in the Netherlands?; (2) To what extent is ambulance personnel able to prevent further escalations during these incidents and how often do they use aggression themselves?; (3) What is the proportion of exposure to potentially traumatic events among ambulance personnel that are aggression‐related? and (4) To what extent do demographics, health, personality, handling of rules, coping and social organizational stressors (independently) predict repeated confrontations with aggression?

### Design

A longitudinal study was undertaken among ambulance personnel using two surveys with a 12‐month interval. There are 25 regional ambulances services (in Dutch: RAV regio's) in the Netherlands. For this study, five regional services were asked to participate (Utrecht, Hollands Midden, Brabant Midden‐West, Brabant Noord, Brabant Zuid‐Oost). One regional service could not participate due to organizational matters. All other services approved the study and cooperated. All participating services first introduced the study among their personnel. Ambulance personnel in the Netherlands consists of nurses and drivers (and some drivers are/were also nurse). Thus, the team on an ambulance car consists of a nurse and driver and this work situation may differ from other countries.

### Participants

Subsequently, all ambulance personnel of the participating services (nurses and drivers) were invited through their work email‐address to participate. Ambulance personnel in the Netherlands consists of nurses and drivers (and some drivers are/were also nurse). Thus, the team on an ambulance car consists of a nurse and driver and this work situation may differ from other countries.

The invitation was accompanied by additional information on our study. In this letter, we did not solely focus on aggression but on potentially traumatic events in general and on coping self‐efficacy. Respondents could participate by filling in a web‐based questionnaire accessible via an attached link. The first survey (T1) took place in 2014 during the spring. Six months after the first survey, the second survey (T2) was conducted in a similar way. At both surveys, reminders were sent to non‐responders. For this type of research, no medical ethical testing committee approval is needed in the Netherlands. However, all respondents gave their electronic informed consent.

### Data collection

A web‐based questionnaire was administered that assessed the following topics at T1: (1) demographics (i.e. gender, age, profession); (2) confrontations with aggression (i.e. physical aggression, serious threat, verbal aggression, being on guard); (3) health and stress symptoms (i.e. anxiety, depression, sleeping problems, general health); (4) personality (agreeableness); (5) handling of rules; (6) coping (seeking support and coping self‐efficacy) and (7) social organizational stressors (problems with colleagues and superiors). Confrontations with aggression were also assessed at T2. Below, the questionnaire will be described in detail.

Confrontations with aggression were assessed, using four questions of the Acute Stress List (van der Velden & Herpers 1994, van der Velden & Kleber 2002, van der Velden *et al*. 2010): (1) ‘How often in the past 12 months have you been confronted with people who used physical aggression against you?’; (2) ‘How often in the past 12 months have you been confronted with people who seriously threatened you (without using physical aggression against you)?’; (3) How often in the past 12 months have you been confronted with people who used verbal aggression aimed at you (without using physical aggression against you)? and (4) ‘How often in the past 12 months have you been confronted with situations you were on guard, because you were afraid that people might become aggressive?’. If respondents were confronted with a form of aggression, sub‐set respondents were automatically and at random asked a follow‐up question ‘In how many cases (of this form of aggression) were you able to prevent further escalation?’ (1 = in most case yes, 2 = sometimes yes, sometimes not, 3 =  most case not).

Confrontations with potentially traumatic events (PTE) in the past 12 months were assessed at T1, using eight fixed answer categories (varying from traffic accidents, violence to suicide; 1 = yes) with the explicit option that respondents could describe another PTE (open answer). We asked respondents to rate/describe which was the most drastic one and how stressful it was for them at the time of the event (1 = not or hardly to 5 = very much). We also asked how often respondents themselves used violence against residents who were aggressive towards the respondent. These items have seven‐point Likert scales indicating the frequency of events (1 = not in past year, 2 = past year once or more, 3 = every 6 months once or more, 4 = every 3 months once of more, 5 = every month once or more, 6 = every week once or more, 7 = each day once or more).

Symptoms of depression (16 items), anxiety (10 items) and sleeping problems (three items) in the 7 days before T1 were assessed using the Symptom Checklist 90‐R (SCL‐90–R, Derogatis 1977). The validity and reliability of the Dutch SCL‐90–R has proven to be satisfactory (Arrindell & Ettema 1986). Items (90) have five‐point Likert scales (1 = not at all to 5 = extremely). All three Cronbachs Alpha's were ≥0·81. In addition, one item from the RAND‐36 assessing general health was added (Aaronson *et al*. 1998) (1 = excellent‐5 = bad).

Agreeableness was measured at T1 using the International Personality Item Pool (IPIP, Goldberg 1999). This scale has 10 items with five‐point likert scales (1 = very inaccurate‐5 = very accurate) and demonstrated strong concurrent validity with other personality measures (Gow *et al*. 2005, Zheng *et al*. 2008). Cronbach's alpha was 0·78. For this study, we developed eight different items, partly based on earlier interviews with ambulance personnel, to assess how respondents perceive how they deal with rules and protocols that might influence behaviour of others (such as prevent or stimulate aggression). The following items were administered at T1: (1) I ‘prefer to adhere to the rules’; (2) I ‘am flexible in applying rules’; (3) I ‘always try to find a solution that is acceptable for all of us’; (4) I ‘think that people must follow my instructions precisely’; (5) I ‘compromise very easily’; (6) I ‘am very capable in handling changing circumstances’; (7) I ‘believe that making people feel satisfied is more important than adherence to rules’; (8) I ‘dislike situations that don't develop as I expected’. All items have the same five‐point Likert scales as the IPIP.

The coping style ‘seeking social support’ with regard to how respondents deal with problems or unpleasant situations, was assessed at T1 using the Dutch UCL (Schreurs 1992). The five items have five‐point Likert scales (1 = seldom or never, 5 = very often). The seven‐item Coping Self‐Efficacy Measure (Bosmans *et al*. 2015) was administered at T1. Respondents rated their perceived capability on dealing with experienced potential traumatic events (see above) and its consequences on seven‐point scale (1 = I'm not at all capable at all‐7 = I'm totally capable). Cronbach's Alpha's were ≥0·80.

Social organizational stressors, i.e. problems with colleagues and superiors (both nine items) were assessed using the Questionnaire on the Experience and Evaluation of Work (QEEW, VVBA in Dutch, van Veldhoven *et al*. 1997, 2002). It has four‐point scales (0 = always, 3 = never) and both Cronbach's Alpha's ≥0·79. As such, our analyses included 19 predictors.

### Ethical considerations

Ambulance personnel of the participating organizations received written and verbal information about the study and respondents gave their electronic/digital informed consent when starting with the online web‐based questionnaire. For this type of research, no medical ethical committee approval is needed in the Netherlands.

### Data analysis

The dependent variable ‘repeated confrontations with aggression’ was defined as follows: being confronted with a specific type of aggression in the year before T1 (answer categories 2‐7) and being confronted with the same type of aggression in the 6 months before T2 (answer categories 3‐7). Based on this definition, for each aggression variable, a separate ‘repeated confrontations with aggression’ was computed. The statistical analyses were conducted in two steps. We first selected relevant variables, i.e. assessed which predictors at T1 were associated with repeated confrontations on a bivariate level of *P* < 0·10 using logistic regression analyses. At step 2, predictors that were associated on a bi‐variate level of *P* < 0·10 were entered simultaneously in multivariate logistic regression analyses. Statistical analyses were performed using SPSS version 22.

## Results

### Non‐response analyses and characteristics

At T1, 213 of 850 persons participated (response = 25·1%) and at the second survey 103 of 213 responded (response = 48·4%). For reasons of privacy, we could not examine to what extent respondents and non‐respondents at T1 differed in demographics or aspects of functioning (f.i. sickness leave): whether or not personnel participated was confidential.

Non‐response analyses at T2 showed minimal differences at T1 between those who only participated at T1 and those who participated at T1 and T2. They did not differ significantly in reported confrontations with aggression, health, personality, handling rules, coping and social organizational stressors. Significantly, more nurses than drivers participated at T2 T1: (53·6% vs. 46·4%) T2: (72·0% vs. 28·0%) (χ^2 ^= 7·527, d.f. = 1, *P = *0·006), but no significant differences between the two occupational functions were found with respect to the dependent variables in this study, i.e. confrontations with aggression. For this reason, function was not included in the list of predictors (a complete list of the descriptives of the study variables can be obtained from the authors).

### Confrontations with aggression

In Table 1, the reported prevalences on confrontations with aggression are presented for both T1 and T2. Table 1 shows that at T1 36 respondents reported being confronted with verbal aggression at least once every 6 months to every month once or more and 33 at T2. In total, 24 of these 36 respondents at T1 (66·6%), also reported these events at T2. With regard to ‘on guard’ these numbers were 42 of 56 (75·0%), indicating that the majority of respondents confronted with these forms of aggression continue to experience these confrontations. Thus, the majority of those reporting aggression incidents at T1, reported similar incidents after T1. Respondents reported that in most cases, they were able to prevent escalation: physical aggression 9 of 9 (100·0%); serious threat 10 of 12 (83·3%); verbal aggression 21 of 28 (75·0%); on guard 39 of 45 (86·7%). According to the plan, the question about being able to prevent further escalation was randomized (25·0%) among the four forms of aggression. Due to the low response rate, the randomization was not precisely 25·0%. In total, seven (6·8%) respondents were, according to our definition, repeatedly confronted with physical aggression, five (4·9%) with serious threat, 32 (31·1%) with verbal aggression and 46 (44·7%) with verbal aggression in this study period. In addition, 54 (52·4%) respondents were repeatedly confronted with any form of aggression (physical or verbal or threat or on guard).

**Table 1:** Frequencies confrontations with aggression and used aggression (*N* = 103)

	Physical aggression		Serious threat		Verbal aggression		On guard for aggression		Used aggression	
	*N*	%	*N*	%	*N*	%	*N*	%	*N*	%
Reported at T1										
Not in past year	59	57·3	62	60·2	26	25·2	8	7·8	83	80·6
Past year once or more	36	35·0	32	31·1	41	39·8	39	37·9	18	17·5
Every 6 months once or more	7	6·8	8	7·8	25	24,3	34	33·0	2	1·9
Every 3 months once or more	1	1·0	1	1·0	7	6·8	13	12·6	–	–
Every 3 months once or more	–	–	–	–	3	2·9	7	6·8	–	–
Every month once or more	–	–	–	–	1	1·0	2	1·9	–	–
Every week once or more	–	–	–	–	–	–	–	–	–	–
Each day once or more	–	–	–	–	–	–	–	–	–	–
Reported at T2										
Not in past year	60	58·3	59	57·3	20	19·4	10	9·7	83	80·6
Past year once or more	35	34·0	36	35·0	50	48·5	45	43·7	16	15·5
Every 6 months once or more	4	3·9	4	3·9	22	21·4	24	23·3	4	3·9
Every 3 months once or more	4	3·9	3	2·9	5	4·9	13	12·6	–	–
Every 3 months once or more	–	–	1	1·0	5	4·9	9	8·7	–	–
Every month once or more	–	–	–	–	1	1·0	2	1·9	–	–
Every week once or more	–	–	–	–	–	–	–	–	–	–
Each day once or more	–	–	–	–	–	–	–	–		

With respect to the most serious potentially traumatic event, in total seven respondents (5·4% of total group and 7·0% of total group excluding three respondents that reported that they were not confronted with any event) reported an event at T1 that can be categorized as a form of aggression towards ambulance personnel in the 12 months before T1 (fixed answer categories: physical aggression *N *=* *2, verbal aggression *N *=* *3, open question: aggression towards colleague *N *=* *1; threatening atmosphere without aggression *N *=* *1). For two of these seven respondents (28·6%) this was rated as ‘much’‐‘very much’ stressful at that time. Examples of other reported events were child reanimation, youngster with a terminal disease, death of colleague and suicide. In total group, 18 of 100 respondents (18·0%) confronted with a PTE reported this level of stress.

### Predictors repeated confrontations

The outcomes of the bi‐variate analyses showed that a limited number of predictors were associated with repeated victimization on a *P *<* *0·10 level. They are presented in Table 2. This table also shows the outcomes of the multivariate regression analyses (due to missing values, *N *=* *95 in these analyses). The results of Table 2 need little explanation. Problems with superiors were the only significant independent predictor for repeated confrontations with verbal aggression. The ability to compromise very easily was the only significant independent predictor of repeatedly being on guard and of the composite variable repeatedly being confronted with any form of aggression. Because of the very low numbers, we excluded repeated physical aggression (*N *=* *7) and repeated serious threat (*N *=* *5) as dependent variables in the logistic regression analyses.

**Table 2:** Results logistic regression analyses (*N* = 95)

	Bi‐variate		Multivariate		
	Odds ratio	*P* value	Adjusted odd ratio	95% confidence interval	*P* value
Predicting repeated verbal aggression					
Gender	2·54	0·05	2·13	(0·81‐6·07)	0·21
Compromise very easily	0·44	0·05	0·55	(0·22‐1·34)	0·19
Health problems	1·69	0·07	1·60	(0·86‐2·99)	0·14
Problems with superior	1·18	0·01	1·16	(1·02‐1·32)	0·02
Predicting repeated on guard					
Gender	2·48	0·05	1·91	(0·74‐4·92)	0·18
Age	0·96	0·09	0·96	(0·91‐1·02)	0·15
Compromise very easily	0·37	0·02	0·39	(0·17‐0·90)	0·03
Predicting repeated any form of aggression					
Gender	2·84	0·03	2·41	(0·91‐6·40)	0·08
Compromise very easily	0·38	0·02	0·41	(0·17‐0·97)	0·04
Health problems	1·59	0·07	1·56	(0·90‐2·71)	0·11
Problems with superior	1·11	0·07	1·08	(0·96‐1·22)	0·19

## Discussion

Aim of the present longitudinal study was to identify predictors of repeated confrontations with aggression among ambulance personnel, i.e. for repeated physical aggression, serious threat, verbal aggression and being on guard because of possible aggression. On the basis of the literature, we assessed the predictive values of demographics, health, personality and dispositional optimism, handling of rules, coping and social organizational stressors. Findings of the multivariate logistic regression analyses showed that problems with superiors were significantly positively associated, while the self‐reported ability to compromise very easily was negatively associated with repeated confrontations, i.e. verbal aggression, being on guard and/or any form of aggression. Our findings of having problems with superior and not being able to easily make a compromise could be seen as factors related to interpersonal conflict (cf. Hogh *et al*. 2005). Being uncompromising towards a patient, obviously has the potential for sparking conflict. Furthermore, problems with one's superior are an interpersonal conflict in itself. Additional analyses showed that being unable to compromise and having problems with a superior were significantly associated, but not strongly at T1 (*R *=* *0·216, *P *=* *0·029). To the best of our knowledge, this is the first study showing the independent predictive value of ability to compromise very easily.

These findings also indicate that the large majority of selected predictors was not significantly associated (not even at a 0·05 ≤ *P *<* *0·10 level) with repeated confrontations. Thus, we found no indications that levels of agreeableness, coping self‐efficacy following PTE, seeking social support as coping style, anxiety symptoms, depression symptoms, sleeping problems, problems with colleagues and other items on handling rules, were associated with a prospective increased risk of repeated confrontations (verbal aggression, being on guard; cf. Magnavita 2014).

Interestingly, a very large proportion of ambulance personnel in our study reported that in most cases they were able to prevent escalations during (potential) aggression incidents. Thus, when looking at these prevalences, we must realize that these numbers are not static and that ambulance personnel are not just/only passive and overwhelmed by these events how drastic they may be. This information indicates that these prevalences could be considered from a different perspective. For example, 75·0% of the respondents faced with verbal aggression said that in most cases they were able to prevent escalations. This indicates that in theory of the 100 verbal aggression incidents, at least between 38‐75 may also be viewed as incidents where respondents did act successfully (prevented escalations). The number of respondents did not enable us to assess the characteristics of this group further, but future research on this topic is definitely warranted to move beyond the prevalences of aggression. Of course, recognition of confrontations with these events and possible adverse effects on mental health such as PTSD‐symptomatology is needed, but recognition of successful interventions of ambulance personnel to prevent escalations is not less essential.

Due to (very) low prevalences of repeated confrontations due to physical aggression (*N *=* *7) and serious threat (*N *=* *5), for statistical reasons, we were unable to assess the predictive values of the aforementioned predictors in our sample (a sample of at least 5‐6 times, our sample should enable these analyses). However, the prevalences of physical aggression, serious threat and being on guard were markedly lower than were found in a study in 2002 among two samples of ambulance personnel (*N *=* *68 and *N *=* *116) using identical questions (62·0‐63·0%, 59·0‐60·0% and 81·5‐91·0% respectively, Grievink *et al*. 2002). Since we found no differences in prevalences of physical aggression, serious threat and being on guard, using identical questions, between different groups of firefighters assessed in 2002 and 2013 (data not shown here) the question arises why we found low prevalences in this study. It may be a result of governmental policies, media‐attention or training programs in the past years to reduce aggression towards rescue workers, but future research is needed to confirm (or reject) this hypothesis.

### Limitations

There are some characteristics and limitations that need to be discussed. Strength of our study is the prospective design. As described in the introduction section, most studies on predictors of confrontations with aggression among nurses are cross‐sectional limiting conclusions: they cannot solve the well‐known ‘egg‐chicken’ problem (cf. Magnavita 2014). In addition, we assessed distinct predictors varying from demographics to social organizational stressors. We used well‐validated and standardized instruments, except for the 8 items on handling rules that we developed for this study. We asked respondents to focus on the past year with respect to aggression: although this timeframe is limited, we cannot completely rule out recall bias as many other studies. Of the five regional ambulance services, four participated and one did not participate for other reasons. However, the response rate at T1 was not high (25·1%) despite all efforts to motivate personnel to participate and our sample is not very large. It is our impression that in the Netherlands, many studies are conducted among ambulance personal (internal as well as external such as ours) introducing the risk of ‘research‐fatique’. We could not conduct a non‐response analysis of the non‐responders at T1, in contrast to the drop‐out analysis at T2. Interestingly, these analyses showed minimal differences between responders and non‐responders at T1. Like all other studies on this topic, we rely on self‐reports as the sole source of information. We did not assess possible internal registration data on such events, reports of the police or observations of for instance superiors and home front, although such data may introduce new problems (such as that many incidents are not systematically reported to superiors, police or home front). Earlier research suggested the existence of peak moments of aggression (Carmel & Hunter 1993). This aspect was not assessed in this study. We did not examine the training history of the respondents that may be associated with a reduced risk of repeated WPV. To analyse this history, we had to ask too many question about (each) received training (year/month of training, all specific elements of training, duration of training, follow‐ups after training, etc.) to be able to interpret findings, since we have no reason/information to assume that received training on WPV was similar across all respondents.

## Conclusions

Nevertheless, our findings suggest that measures (training, procedures, rules) to prevent repeated verbal aggression and being on guard, should especially target facilitating or improving the ability to compromise very easily and diminishing or solve problems with superiors. This study also found that the proportion of potentially traumatic events due to aggression is relatively limited: seven respondents reported an aggression incident in this perspective of which two rated this events as ‘much’ or ‘very much’ stressful. In other words, although many respondents are confronted with one of more forms of aggression, most reported potentially traumatic events are not related to aggression. To the best of our knowledge, no studies are available among ambulance personnel (or comparable groups) that did focus on the prevalences of perceived successful interventions during such events. Interestingly, many respondents reported that in most cases they were able to prevent further escalations. Since the numbers were relatively low, more research on this interesting topic is warranted. In addition, it would be very interesting to examine to what extent the ability to prevent escalations is related to finding in this study that the large majority of potentially traumatic events were not aggression‐related. Based on the definition/criteria of PTSD (DSM‐IV), where feelings of helplessness were explicitly mentioned to describe traumatic events, one might hypothesize that there is a positive relation between both; the perceived ability to prevent escalations may reduce feelings of helplessness. In any way, findings suggest that ambulance personnel should not only be viewed as ‘victims’ but also should receive recognition for their apparently successful interventions to prevent escalations during WPV.

## Conflict of interest

No conflict of interest has been declared by the authors.

## Author contributions

All authors have agreed on the final version and meet at least one of the following criteria [recommended by the IC‐MJE (http://www.icmje.org/ethical_1author.html)]:

- substantial contributions to conception and design, acquisition of data, or analysis and interpretation of data;
- drafting the article or revising it critically for important intellectual content.
